# Annotations

**Published:** 1888-01-28

**Authors:** 


### Annotations.

"Fish as "Brain Food."

"Fish," says Dr. W. W. Godding, of Washington, "has for years enjoyed the reputation of being rich in phosphorus, and hence adapted to the growth of brains. How such a notion originated I do not know; perhaps, because stale fish shines with a phosphorescent light in the dark. As a food, fish is richer in water than in phosphorus, and to feed it to children, expecting thereby to grow them into philosophers, would be on a par with the scholar who boiled his dictionary in milk for supper, hoping thus to acquire the language. The dwellers by the sea—the Esquimaux, the fishermen of Labrador and Cape Cod —have not shown any preponderance of brain. . . I am not condemning fish as a food in health or disease. Easily digested, it is often a very suitable food, but not on account of its phosphorus. The world could ill afford to do without its oysters; and there is hardly anything more delicious than a freshly-caught trout, especially if you have been following the brook for hours to catch him." The shrewdness of the American physician is well seen in the observation that the fishermen of Labrador and Cape Cod have never been famous for brains. It is worth while to take an occasional note of what is now transpiring, and of what has long been going on in the great world's physiological laboratory. The remark about the Cape Cod men, leads naturally to the inquiry, are fishermen as a class men of unusual brains, or have they ever been so in the history of the world? We are not aware of any statistics or other data which would enable us to answer this question with any approach to scientific precision. But falling back on remembered experience, we are inclined to think that fishermen as a class occupy a relatively low place in the intellectual scale. Courage and endurance they have in abundance; but it is probable that the proportion of those who pass from their ranks to those of the more intellectual classes, is smaller than that from any other manual calling. These physiological experiments, so often repeated on a large scale, rather negative the popular idea of the value of fish as a "brain food."

A Plea for the Insane.

Dr. G. A. Tucker, proprietor of a private lunatic asylum in Sydney, Australia, recently made a tour of inspection among institutions for the insane in Europe and North America, which is perhaps without parallel in history. In all he travelled about 140,000 miles and visited over 400 asylums at his own cost. The fact of his undertaking such a series of expeditions and of his carrying them all out with such completeness argues an ardour and a zeal in the pursuit of special knowledge which are beyond all praise. It is impossible even to indicate, in the space at disposal, the varied contents of Dr. Tucker's book, published after his exhaustive inquiries. On the subject of the visitation of patients in asylums he makes some observations which the relatives and friends of the insane, as well as all those who may have any concern with them, should lay seriously to heart. "Generally speaking," says Dr. Tucker, "the official visitation of asylums is open to three objections: (1) The visits are not frequent enough; (2) they are made (only) at stated times; (3) the inspection is not sufficiently minute. In my opinion non-official visitation and inspection are at least equally important with the official. Asylums should be more freely open to inspection by the medical profession and the public of all classes. They should *at all reasonable times* be accessible to friends of patients and representatives of the press. The hall-porter should keep a register of all persons visiting the patients, stating whether they have been seen or not, and if not, giving the reasons why. The visitation, or outside supervision, in all parts of the world, is not adequate to the necessity which exists for it. The law in most countries enforces the residence of the insane within the walls of the asylum for the protection of the general public, and that being so, the law should also take care to surround the persons it thus confines with the largest possible guaranty for that care and proper treatment which their helpless condition and incapacity for self-protection require. But it is too often the case that the patient has little or no protection on taking up his residence among strangers, and in many instances he is soon abandoned by his nearest friends, only because of the cost and difficulty of access to the asylum, or from the belief and assurance that there is no hope of recovery. Under these circumstances it is no wonder that patients settle down into a state of chronic insanity: the mind works on itself for months, hoping for the freedom which never comes. It is absolutely necessary that insane people should have someone to talk to." These are the carefully-weighed utterances of a specialist of the largest knowledge and the most unique experience, and should be weighed and remembered accordingly.

"Egg-Phosphate" for overstrained nerves.

Our American cousins are great at all kinds of remedies demanded and dictated rather by popular taste than by physiological fitness or necessity. "Brandy cock-tails," "Eye-openers," and even "Corpse-revivers," are familiar institutions on "the other side." The last thing of the kind is perhaps "Egg-phosphate," whatever that may be, which is said to be a "panacea for overstrained nerves and exhausted brains." The compound seems to consist of whisked eggs, new-laid of course, and an effervescent drink such as the exhausted one may himself choose. In the case of the man who likes "a little wine for his stomach's sake," champagne may be the vehicle; whilst the followers of Mr. J. B. Gough will doubtless select soda water. The phosphorus contained in the egg is said to be the active agent in reviving the exhausted brains; on the same principle, we suppose, that it is the lemon and sugar in the toddy, and not the whisky, which upsets the liver. The American physicians do not seem to be impressed by the preternatural sharpness of the druggists and saloon proprietors. "The search for health," says Dr. Godding, "through the icy draughts of a soda fountain is illusive. If the stomach were made less a beast of burden there would be fewer dyspeptics and sounder brains. What the brain of this busy, pushing age wants is rest and not phosphorus." This very decided and very sound philosophy may be commended to all those who have wit enough to see and appreciate its value. Lord Grimthorpe notwithstanding, it is sometimes wise to pay a little heed to what the doctors say.

Well-paid Science.

The chair of Botany at the University of Edinburgh is now vacant by the death of Professor Alexander Dickson, M.D. This chair is one of the ripest plums which hang on the tree of botanical science, and will probably be eagerly sought after by some of the most eminent botanists in Great Britain. It is said to be worth £3,000 a year in money, in addition to a handsome mansion in the Arboretum, rent free. When it is further stated that the Arboretum is a large and beautifully wooded park in one of the finest situations in the neighbourhood of Edinburgh, it is easily seen that the post is a highly enviable one. The botany and zoology classes at the Scotch Universities are held in the summer, and the session is only three months long. A thousand pounds a month and a mansion in a fine park are not prizes open to botanists every day, and it may be anticipated that there will be such a choice of applicants as will enable the University Senate to appoint a fit successor to the amiable and distinguished professor whose too early death has caused the vacancy. While on this topic it may be useful to remind the Senate and other educational authorities that the best way to make successful students and practitioners is to give them plenty of thoroughly competent teachers. Might not a third of the £3,000 a-year be devoted to the payment of four capable assistants, and might not something be done in the way of lecturing and teaching during the winter months?

